# The Effect of Social Information Processing, Self-Regulation and Metacognition on Theory of Mind: Path Analysis †

**DOI:** 10.3390/brainsci14090943

**Published:** 2024-09-21

**Authors:** Canan Keleş Ertürk, Kezban Tepeli

**Affiliations:** 1 Child Development Program, Vocational School of Health Services, KTO Karatay University, 42020 Konya, Turkey; 2 Child Development, Faculty of Health Sciences, Selcuk University, 42250 Konya, Turkey

**Keywords:** theory of mind, metacognition, social information processing, emotion processing, self-regulation

## Abstract

Objectives: The main purpose of the current study was to examine the effects of social information processing, self-regulation, and metacognition variables on ToM using path analysis. Methods: For this purpose, path analysis was conducted for the model established according to the relationships between the variables. Theory of mind task battery (ToMTB), Metacognitive Knowledge Interview (McKI), Schultz Test of Emotion Processing—Preliminary Version (STEP-P), and self-regulation scale were administered to 310 children aged 3–5 years. Results: The results show that social information processing (except STEP-P.P.B, one of the sub-dimensions of the STEP-P scale) has a significant effect on ToM through metacognition and self-regulation. Conclusions: In this context, it can be said that social information processing, metacognition, and self-regulation are effective in the development of ToM.

## 1. Introduction

### 1.1. Literature Review

Theory of mind, defined as the ability to predict and explain other people’s behavior, is considered an important step in social and cognitive development [1]. Theory of mind is the ability to connect mental states both to oneself and to others. These mental states can include beliefs, desires, tensions, and emotions. However, it is not limited to these [2]. Theory of mind is the basis of understanding human behavior. The theory of mind takes into account mental states in the process of explaining, predicting, and interpreting people’s behavior. In the process of social interaction, there is a need for explainers, interpretations, and predictions regarding behavior. In this context, ToM constitutes an important part of social understanding and social cognition in people’s lives [3]. Theory of mind is usually acquired around four in children with typical development and progresses gradually. However, it is observed that children with autism spectrum disorder, who have problems, especially in communication and social skills, also experience difficulties and delays in ToM skills [4]. ToM has significant differences in the brain development of individuals with autism spectrum disorder and children with typical development. Typically developing children start to build ToM skills at the age of 4–5 years. This development process is supported by the maturation of brain regions such as the prefrontal cortex, temporoparietal junction (TPJ), and superior temporal sulcus (STS). The normal development of these brain regions enables children to understand the minds of others. However, in individuals with autism spectrum disorder (ASD), structural and functional differences are observed in these brain regions. Abnormalities in the function of TPJ and the prefrontal cortex cause individuals with autism to have difficulties in interpreting social cues and understanding the perspectives of others [5].

A related area that partially overlaps with the theory of mind is the development of metacognition, which concerns the child’s knowledge of the variables of the person, task, and strategy involved in the mastery of cognitive tasks and their ability to monitor and control their cognitive processes. Metacognition focuses primarily on cognitive acquisition (e.g., memorization), the contributions of metacognitive knowledge, and metacognitive monitoring, while the theory of mind is concerned with the conceptual underpinnings of these abilities [6]. Metacognition, which is associated with cognitive and social development, is often referred to as a conscious and deliberate mental activity [7]. Regarding the assessment of metacognition in children, Garner and Alexander (1989) suggest three ways to do this. These include asking children directly about their metacognition, having children think aloud while doing a task and asking children to come up with a good solution for a young child’s problem [8]. Self-regulation refers to planned and cyclically adapted thoughts, feelings, and actions to achieve personal goals. This definition differs from definitions that emphasize a singular trait, ability, or stage of competence. Self-regulation is described as cyclical because feedback from previous performance is used to make adjustments during current efforts [9].

The social information processing model is a model proposed to explain how behavioral responses are affected by mental processing in social interaction settings [10]. The social information processing model provides a comprehensive model of how children process, interpret, and make decisions about cues in social situations [11]. This model recognizes that children enter social situations with preexisting influences, both biological and environmental [12]. Supporting children’s social information processing skills from early childhood can help them develop their social behaviors during school years and positively affect their academic success. However, deficiencies in social information processing can put children at a disadvantage in the classroom. This can result from children misinterpreting the behaviors of others and their inadequate social behaviors [13].

When the studies on ToM in the literature are examined, studies examining the relationship between ToM and language skills, verbal abilities, child–peer interaction, social interaction, social–moral competence, inhibitory control, and empathy attract attention [14,15,16,17,18,19,20,21,22,23,24,25,26,27]. However, it is also seen that studies on the determinants of metacognition [28,29], self-regulation [30], and social information processing [31,32] on the theory of mind are limited in the literature. The view that the capacity to think about the theory of mind is closely related to the development of metacognition, self-regulation, and social understanding is taken as the basis of this study [33]. Therefore, in this study, the effect of metacognition, social information processing, and self-regulation on the theory was examined.

### 1.2. Research Questions

This study aimed to test the hypothesis that “social information processing significantly affects the theory of mind through metacognition and self-regulation.” In line with the general hypothesis, the following hypotheses were tested:

**H1.**  *Social information processing significantly affects the theory of mind through metacognition.*

**H2.**  *Social information processing significantly affects the theory of mind through self-regulation.*

**H3.**  *Social information processing significantly affects the theory of mind through metacognition and self-regulation.*

**Figure 1:**
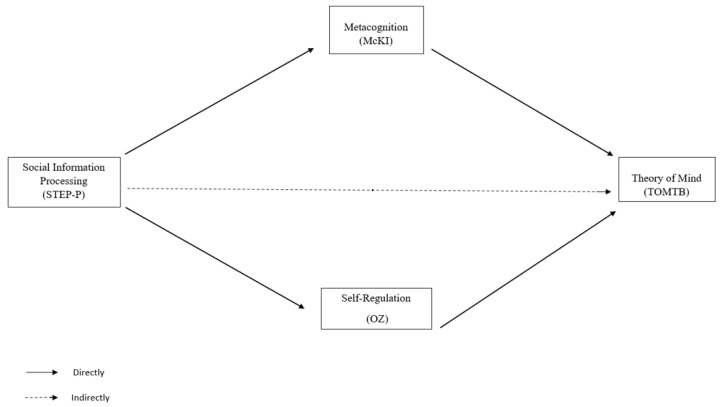
Theoretical model.

The theoretical model for this study is shown in Figure 1.

## 2. Method

This study was conducted by scientific ethical principles. The ethics committee approved the research, and research and publication ethics were complied with. Before the implementation, permission was obtained from the Ministry of National Education, and data were collected by the researcher in the 2020–2021 (1 September 2020–18 June 2021) academic year. Written informed-consent forms were obtained from the participants’ parents.

### 2.1. Participants

A general survey model has been used in this research. The sample of the study was determined by the Appropriate Case Study Group, one of the Purposeful Study Groups. The Appropriate Case Study Group is the selection of individuals and groups on which research will be conducted easily [34] (p. 175). Based on this, five preschools were identified. The study group of this research consists of a total of 310 children (3–5 age group) who attend the kindergarten under the Ministry of National Education, in the city center of Konya. In studies on factor analysis, the sample size is generally calculated as five times the number of items [35]. Based on previous studies, it was determined that the sample size of 310 provided sufficient power to detect direct and indirect effects [36,37]. In particular, we used a simulation-based power analysis method for regression-based mediation [38]. We found that our sample size was large enough to detect direct and indirect effects. Therefore, the sample in this study was determined as 310 people. This study was conducted by scientific ethical principles. Before the implementation, permission was obtained from the Ministry of National Education, and data were collected by the researcher in the 2020–2021 (1 September 2020–18 June 2021) academic year. Written informed consent forms were obtained from the participants’ parents. Descriptive statistics on the personal characteristics of the participants are presented in Table 1, and descriptive statistics on the characteristics of the participants’ parents are presented in Table 2.

Table 1 shows that the average age of the children of the parents who participated in the study was 58 months. Of the children, 151 (48.7%) were girls, and 159 (51.3%) were boys. In total, 160 (51.6%) were first-born children, and 72 (23.2%) were only-children. The duration of school attendance was less than 6 months in 161 (51.9%) children. Considering the socio-economic level of the families, 8 (2.6%) had a low economic level, 272 (87.7%) had a medium economic level, and 30 (9.7%) had a high economic level.

Table 2 shows that while the mothers of 70 (22.6%) children are 29 years old or younger, there are 30 (9.7%) children whose fathers are 29 years old and younger. There are 25 (8.1%) children whose mothers have postgraduate degrees and 50 (16.1%) children whose fathers have postgraduate degrees. In addition, there are 162 (52.3%) children whose mothers are working and 304 (98.1%) whose fathers are working.

### 2.2. Materials

A general information form, theory of mind task battery (TOMTB), Metacognitive Knowledge Interview (McKI), Schultz Test of Emotion Processing—Preliminary Version (STEP-P), and Preschool Self-Regulation Assessment were used as data collection tools in the study.

*General Information Form:* The *“General Information Form”* is a multiple-choice form prepared by researchers to determine the demographic characteristics of the participants.

*Theory of Mind Task Battery (TOMTB):* The theory of mind task battery was developed by Hutchins et al. The development study of the original form of TOMTB was conducted with both typically developing children and children with autism spectrum disorders, and validity and reliability studies were conducted for both groups. It aims to assess the ToM skills of children. Consisting of 15 items, the TOMTB is applied by conveying questions with pictures and obtaining the answers from the pictures. The answers given by the children are scored as 1 (correct) and 0 (incorrect) [39].

*Metacognitive Knowledge Interview (McKI):* Developed by Marulis et al. (2016), it is an interview form developed to examine the meta-cognitive knowledge of children aged 3–5. Children are first given tasks using the Wedgits material. Then, they move on to the 15-item interview form. The answers given by the children are evaluated as 0, 1, and 2 points [40].

*Schultz Test of Emotion Processing—Preliminary Version (STEP-P):* STEP-P was developed by Schultz et al. It is a test consisting of video and interview forms developed to examine the social information processing skills of 3–5-year-old children. STEP-P consists of 62 items and 3 main sub-dimensions. The Emotions Subtest has 2 dimensions. The Emotion Recognition dimension (STEP-P.D1) is related to choosing the right emotion in the sketch scenarios in the video. The Anger Attribution Dimension (STEP-P.D2) consists of the choice of the expression “angry” as an emotion. The Goal Acquisition Subtest has 3 dimensions. The Positive Evaluation of Collaboration Dimension (STEP-P.H1) is related to the child’s use of collaborative behaviors, attitudes, and strategies to achieve their goal. The Positive Evaluation of Aggression Dimension (STEP-P.H2) is concerned with the child’s use of aggressive behaviors, attitudes, and strategies to achieve their goal. The Moral Acceptability of Aggression Dimension (STEP-P.H3) is related to the moral acceptability of the selected aggressive behavior. The Provocation Subtest has 4 dimensions. The A dimension (STEP-P.P.A) is related to the reasons for the behavior, the B dimension (STEP-P.P.B) is related to the interpretation of the behavior as being rude or a good reason or knowingly or accidentally, and the C dimension (STEP-P.P.C) is related to the emotion that the behavior will make the person feel; the D (STEP-P.P.D) and E (STEP-P.P.E) dimensions are related to the response from the children regarding the expected behaviors and reactions depending on the scenario [41].

*Preschool Self-Regulation Scale:* The preschool self-regulation scale was developed to assess self-regulation in the emotional, attention, and behavioral domains using a short, structured set of tasks for children aged 41–70 months. The Turkish adaptation study was carried out by Fındık Tanrıbuyurdu (2012) with preschool children. The scale consists of two parts, the practitioner’s guide and the practitioner evaluation form, and a total of 16 items and 9 tasks. The maximum score that children can obtain from this scale is 48 [42].

### 2.3. Procedure and Data Analysis

First of all, permission was obtained from the developers of the measurement tools. Then, translation and reverse translation procedures were applied for the translation of the TOMTB, McKI, and STEP-P from English to Turkish, and language equivalence was ensured. Then, field experts were consulted to evaluate the content of the tools.

Before the implementation, permission was obtained from the Ministry of National Education, and data were collected by the researcher in the 2020–2021 academic year. The implementation of the 10-item theory of mind task battery took approximately 15 min; the implementation of the 62-item STEP-P test took approximately 70 min; the implementation of the 15-item MCKI took approximately 30 min; and the implementation of the 9-item Preschool Self-Regulation scale took approximately 30 min. The completion of the application takes approximately 145 min for each child. Therefore, considering the attention spans of preschool children, the measurement tools were administered on consecutive days based on subdimensions. In the study, data were collected through face-to-face and individual interviews. The test–retests were collected during the academic year for which permission was obtained.

Before starting the basic analysis, the prerequisites of the structural equation model such as sample size, outlier analysis, multicollinearity problem, and normality assumption were examined [43]. After examining the suitability of the data, the bootstrap method will be used to test the mediation models instead of Baron and Kenny’s (1986) traditional method and Sobel test. The Bootstrap method allows for modeling a large number of regression equations by resampling from the original data and increases the reliability of the results [44,45]. In this study, the indirect effects of mediator variables are evaluated using the bootstrap technique over a sample of 5000 individuals. Non-zero values in the 95% confidence interval of the mediator variables indicate that the indirect effect is significant. Therefore, the bootstrap method will be preferred in testing mediation models since it provides more reliable results [46]. *p* < 0.05 level is considered statistically significant.

## 3. Results

In this study, the relationship between social information processing, metacognition, and self-regulation with theory of mind has been examined by path analysis, which is one of the structural equation modeling types. According to Alpar (2011), path analysis includes the stages of creating a path diagram to show the relationship between variables, determining the amount and direction of the linear relationship, investigating the effects of the relationship (direct and indirect), and interpreting these relationships [47] (p. 1034).

When Model 1 is analyzed in Table 3, a one-unit increase in STEP-P.D1 scores increases TOMTB scores by 0.888 units. STEP-P.D1 scores explain TOMTB scores by 7%. The model is statistically significant (*p* < 0.05). A one-unit increase in STEP-P.D1 scores increases MCKI scores by 0.262 units. STEP-P.D1 scores explain MCKI scores by 3%. The model is statistically significant (*p* < 0.05). A one-unit increase in STEP-P.D1 scores increases OZ scores by 1.162 units. STEP-P.D1 scores explain OZ scores by 18%. The model is statistically significant (*p* < 0.05). In the multiple regression model for the effect of the STEP-P.D1, MCKI, and OZ scores on TOMTB scores, a one-unit increase in STEP-P.D1 scores increases TOMTB scores by 0.480 units. A one-unit increase in MCKI scores increases STEP-P.D1 scores by 0.686 units. A one-unit increase in OZ scores increases STEP-P.D1 scores by 0.196 units. STEP-P.D1, MCKI and OZ scores explain TOMTB scores by 21%. The model is statistically significant (*p* < 0.05).

When Model 2 is analyzed in Table 3, a one-unit increase in STEP-P.D2 scores decreases TOMTB scores by 1.084 units. STEP-P.D2 scores explain TOMTB scores by 8%. The model is statistically significant (*p* < 0.05). A one-unit increase in STEP-P.D2 scores decreases MCKI scores by 0.325 units. STEP-P.D2 scores explain MCKI scores by 4%. The model is statistically significant (*p* < 0.05). A one-unit increase in STEP-P.D2 scores decreases OZ scores by 1.586 units. STEP-P.D2 scores explain the OZ scores by 28%. The model is statistically significant (*p* < 0.05). In the multiple regression model for the effect of STEP-P.D2, MCKI and OZ scores on TOMTB scores, one-unit increase in STEP-P.D2 scores decreases TOMTB scores by 0.601 units. A one-unit increase in MCKI scores increases TOMTB scores by 0.689 units. A one-unit increase in OZ scores increases TOMTB scores by 0.163 units. STEP-P.D2, MCKI and OZ scores explain TOMTB scores by 21%. The model is statistically significant (*p* < 0.05).

When Model 3 is analyzed in Table 3, one-unit increase in STEP-P.H1 scores increases TOMTB scores by 1.840 units. STEP-P.H1 scores explain TOMTB scores by 17%. The model is statistically significant (*p* < 0.05). A one-unit increase in STEP-P.H1 scores increases MCKI scores by 0.817 units. STEP-P.H1 scores explain MCKI scores by 16%. The model is statistically significant (*p* < 0.05). A one-unit increase in STEP-P.H1 scores increases OZ scores by 0.847 units. STEP-P.H1 scores explain OZ scores by 6%. The model is statistically significant (*p* < 0.05). In the multiple regression model for the effect of STEP-P.H1, MCKI and OZ scores on TOMTB scores, one-unit increase in STEP-P.H1 scores increases TOMTB scores by 1.260 units. A one-unit increase in MCKI scores increases TOMTB scores by 0.478 units. A one-unit increase in OZ scores increases TOMTB scores by 0.223 units. STEP-P.H1, MCKI and OZ scores explain 26% of TOMTB scores. The model is statistically significant (*p* < 0.05).

When Model 4 is analyzed in Table 3, a one-unit increase in STEP-P.H2 scores decreases TOMTB scores by 0.632 units. STEP-P.H2 scores explain TOMTB scores by 2%. The model is statistically significant (*p* < 0.05). A one-unit increase in STEP-P.H2 scores decreases MCKI scores by 0.374 units. STEP-P.H2 scores explain MCKI scores by 3%. The model is statistically significant (*p* < 0.05). The effect of STEP-P.H2 scores on OZ scores was not statistically significant (*p* > 0.05). In the multiple regression model for the effect of STEP-P.H2, MCKI and OZ scores on TOMTB scores, the effect of STEP-P.H2 scores on TOMTB scores was not statistically significant. A one-unit increase in MCKI scores increases TOMTB scores by 0.670 units. A one-unit increase in OZ scores increases TOMTB scores by 0.273 units. STEP-P.H2, MCKI and OZ scores explain TOMTB scores by 20%. The model is statistically significant (*p* < 0.05).

When Model 5 is analyzed in Table 3, a one-unit increase in STEP-P.H3 scores decreases TOMTB scores by 1.486 units. STEP-P.H3 scores explain TOMTB scores by 4%. The model is statistically significant (*p* < 0.05). A one-unit increase in STEP-P.H3 scores decreases MCKI scores by 1.055 units. STEP-P.H3 scores explain MCKI scores by 10%. The model is statistically significant (*p* < 0.05). A one-unit increase in STEP-P.H3 scores decreases OZ scores by 1.004 units. STEP-P.H3 scores explain OZ scores by 3%. The model is statistically significant (*p* < 0.05). In the multiple regression model for the effect of STEP-P.H3, MCKI and OZ scores on TOMTB scores, the effect of STEP-P.H3 scores on TOMTB scores was not statistically significant. A one-unit increase in STEP-P.H3 MCKI scores increases TOMTB scores by 0.652 units. A one-unit increase in OZ scores increases TOMTB scores by 0.261 units. STEP-P.H3, MCKI, and OZ scores explain TOMTB scores by 20%. The model is statistically significant (*p* < 0.05).

When Model 6 is analyzed in Table 3, a one-unit increases in STEP-P.P.A score increases TOMTB scores by 1.552 units. STEP-P.P. scores explain TOMTB scores by 12%. The model is statistically significant (*p* < 0.05). A one-unit increase in STEP-P.P.A score increases MCKI scores by 0.691 units. STEP-P.P. scores explain MCKI scores by 11%. The model is statistically significant (*p* < 0.05). A one-unit increase in STEP-P.P.A score increases OZ scores by 1.078 units. STEP-P.P. scores explain OZ scores by 9%. The model is statistically significant (*p* < 0.05). In the multiple regression model for the effect of STEP-P.P.A, MCKI, and OZ scores on TOMTB scores, a one-unit increase in STEP-P.P.A score increases TOMTB scores by 0.923 units. A one-unit increase in MCKI scores increases STEP-P.P. scores by 0.579 units. A one-unit increase in OZ scores increases STEP-P.P. scores by 0.212 units. STEP-P.P.A, MCKI, and OZ scores explain 23% of TOMTB scores. The model is statistically significant (*p* < 0.05).

When Model 7 is analyzed in Table 3, a one-unit increase in STEP-P.P.B scores decreases TOMTB scores by 0.555 units. STEP-P.P.B scores explain TOMTB scores by 3%. The model is statistically significant (*p* < 0.05). The effect of STEP-P.P.B scores on MCKI scores was not statistically significant (*p* > 0.05). The effect of STEP-P.P.B scores on OZ scores was not statistically significant (*p* > 0.05). In the multiple regression model for the effect of STEP-P.P.B, MCKI, and OZ scores on TOMTB scores, a one-unit increase in STEP-P.P.B scores decreases TOMTB scores by 0.460 units. A one-unit increase in MCKI scores increases TOMTB scores by 0.671 units. A one-unit increase in OZ scores increases TOMTB scores by 0.270 units. STEP-P.P.B, MCKI, and OZ scores explain 22% of TOMTB scores. The model is statistically significant (*p* < 0.05).

When Model 8 is analyzed in Table 3, a one-unit increase in STEP-P.P.C scores increases TOMTB scores by 1.040 units. STEP-P.P.C scores explain TOMTB scores by 8%. The model is statistically significant (*p* < 0.05). A one-unit increase in STEP-P.P.C scores increases MCKI scores by 0.580 units. STEP-P.P.C scores explain MCKI scores by 11%. The model is statistically significant (*p* < 0.05). A one-unit increase in STEP-P.P.C scores increases OZ scores by 0.574 units. STEP-P.P.C scores explain OZ scores by 4%. The model is statistically significant (*p* < 0.05). In the multiple regression model for the effect of STEP-P.P.C, MCKI, and OZ scores on TOMTB scores, a one-unit increase in STEP-P.P.C scores increases TOMTB scores by 0.549 units. A one-unit increase in MCKI scores increases TOMTB scores by 0.599 units. A one-unit increase in OZ scores increases TOMTB scores by 0.251 units. STEP-P.P.C, MCKI, and OZ scores explain 21% of TOMTB scores. The model is statistically significant (*p* < 0.05).

When Model 9 is analyzed in Table 3, a one-unit increase in STEP-P.P.D scores increases TOMTB scores by 1.963 units. STEP-P.P.D scores explain TOMTB scores by 19%. The model is statistically significant (*p* < 0.05). A one-unit increase in STEP-P.P.D scores increases MCKI scores by 0.531 units. STEP-P.P.D scores explain MCKI scores by 6%. The model is statistically significant (*p* < 0.05). A one-unit increase in STEP-P.P.D scores increases OZ scores by 0.945 units. STEP-P.P.D scores explain OZ scores by 7%. The model is statistically significant (*p* < 0.05). In the multiple regression model for the effect of STEP-P.P.D, MCKI, and OZ scores on TOMTB scores, a one-unit increase in STEP-P.P.D scores increases TOMTB scores by 1.489 units. A one-unit increase in MCKI scores increases TOMTB scores by 0.563 units. A one-unit increase in OZ scores increases TOMTB scores by 0.185 units. STEP-P.P.D, MCKI, and OZ scores explain 29% of TOMTB scores. The model is statistically significant (*p* < 0.05).

When the direct effect is analyzed in the mediation model in Table 4, STEP-P.D1 scores increase TOMTB scores by 0.480; MCKI scores increase TOMTB scores by 0.180; OZ scores increase TOMTB scores by 0.228; MCKI and OZ scores increase TOMTB scores by 0.408 units (*p* < 0.05).

When the direct effect is analyzed in the mediator model, STEP-P.D2 scores decrease TOMTB scores by 0.601 units and TOMTB scores by 0.224 units with the mediating effect of MCKI scores. There is no mediating effect of OZ scores on the effect of STEP-P.D2 scores on TOMTB scores. STEP-P.D2 scores decrease TOMTB scores by 0.483 units with the mediating effect of MCKI and OZ scores (*p* < 0.05).

When the direct effect is analyzed in the mediator model, STEP-P.H1 scores increase TOMTB scores by 1.260 units. STEP-P.H1 scores increase TOMTB scores by 0.390 units with the mediating effect of MCKI scores. STEP-P.H1 scores increase TOMTB scores by 0.189 units with the mediating effect of OZ scores. STEP-P.H1 scores increase TOMTB scores by 0.579 units with the mediating effect of MCKI and OZ scores (*p* < 0.05).

When the direct effect is analyzed in the mediator model, STEP-P.H2 scores do not affect TOMTB scores. STEP-P.H2 scores decrease TOMTB scores by 0.250 units with the mediating effect of MCKI scores. There is no mediating effect of OZ scores on the effect of STEP-P.H2 scores on TOMTB scores. STEP-P.H2 scores decrease TOMTB scores by 0.261 units with the mediating effect of MCKI and OZ scores (*p* < 0.05).

When the direct effect is analyzed in the mediator model, STEP-P.H3 scores do not affect TOMTB scores. STEP-P.H3 scores decrease TOMTB scores by 0.687 units with the mediating effect of MCKI scores. STEP-P.H3 scores decrease TOMTB scores by 0.261 units with the mediating effect of OZ scores. STEP-P.H3 scores decrease TOMTB scores by 0.950 units with the mediating effect of MCKI and OZ scores (*p* < 0.05).

When the direct effect was analyzed in the mediator model, STEP-P.P. scores increased TOMTB scores by 0.923; the mediating effect of MCKI scores increased TOMTB scores by 0.400; the mediating effect of OZ scores increased TOMTB scores by 0.229; the mediating effect of MCKI and OZ scores increased TOMTB scores by 0.629 units (*p* < 0.05).

When the direct effect was analyzed in the mediator model, STEP-P.P.B scores decreased TOMTB scores by 0.460 units. MCKI and OZ scores had no mediating effect on the effect of STEP-P.P.B scores on TOMTB scores (*p* > 0.05).

When the direct effect was analyzed in the mediator model, STEP-P.P.C scores, increased TOMTB scores by 0.549 units; mediating effect of MCKI scores increased TOMTB scores by 0.347 units; mediating effect of OZ scores increased TOMTB scores by 0.144 units; mediating effect of MCKI and OZ scores increased TOMTB scores by 0.491 units (*p* < 0.05).

When the direct effect was analyzed in the mediator model, STEP-P.P.D scores increased TOMTB scores by 1.489; the mediating effect of MCKI scores increased TOMTB scores by 0.299; TOMTB scores increase by 0.175 with the mediating effect of OZ scores, and TOMTB scores increase by 0.474 units with the mediating effect of MCKI and OZ scores (*p* < 0.05).

## 4. Discussion

Based on the view that the thinking capacity related to the theory of mind is closely related to the development of metacognition, self-regulation, and social understanding [33] (p. 71), in this study, the effect of metacognition, social information processing, and self-regulation variables on the theory of mind was revealed by path analysis, one of the types of structural equation modeling.

**H1.**  *Social information processing significantly affects the theory of mind through metacognition.*

In this study, it was found that social information processing significantly affects the theory of mind through metacognition. Metacognition, which is considered as a mediator in the effect of social information processing on theory of mind, is closely related to social information processing. Robson (2006) and Downing et al. (2007) drew attention to the close relationship between social context and metacognition. Again, the theoretical perspective focused on the role of social factors rather than the child’s active contribution to promoting metacognitive development [33,48]. These social factors focus on parents, teachers, and peers in the interaction process. In particular, metacognitive skills are thought to be developed and facilitated through social interaction [49,50]. How individuals think about their thinking and how they develop metacognition about themselves, others, tasks, and strategies depends on the social and cultural context [51] (pp. 13–14). Peer education and other forms of peer-supported learning positively affect metacognition [52], and the importance of social context in promoting the effective use of metacognitive tools is emphasized [53]. Consistent with the literature on the effect of social factors on the development of metacognition [48,50,51,54,55], the results of this study show that social information processing has a significant effect on metacognition. In the process of social information processing, the child’s focus on behavioral reasons and focusing on the emotion that the child is likely to feel affect metacognition. One aspect of metacognition involves awareness of one’s emotions [56]; the other aspect involves monitoring the emotions and actions of group members in social environments and choosing solution strategies [57].

Accordingly, the variables and previous experiences in the social environment in which the individual enables them to understand other individuals, make sense of environmental cues, and thus decide on a response or behavior. Then, they realize their thoughts through self-orientation. Considering that human beings are social beings, the necessity of social information processing for metacognition development also emerges. Metacognition also develops through social learning. Especially at a young age, activities and conversations with families enable children to understand words such as interpretation, thinking, etc., and to develop memory and imagination, so that the development of metacognition can be supported by social contexts. Social environments that allow children to relax and have fun can also increase these gains.

**H2.**  *Social information processing significantly affects the theory of mind through self-regulation.*

In the study, it was found that social information processing significantly affects the theory of mind through self-regulation. Children learn self-regulation skills by observing models and the results of their behaviors, how the model expresses strategies to become better, and how they evaluate and react to their performance [58]. The social information processing model includes the processing of social events, attributions of problems encountered, and decisions. In social-cognitive models, schemas that include beliefs about the consequences of certain types of behavior are associated with outcome expectations, and these beliefs play a central role in determining the solution that the child decides to implement in the next steps of information processing. Children’s expectations about the consequences of their behaviors serve an important self-regulation role in controlling their aggressive behaviors [59]. In the social information processing process, children monitor the impact of their behavior on others involved in the social interaction, and when the behavior does not lead to the desired effect, it is modified accordingly. This is related to the process of self-regulation. This process of self-regulation involves the individual’s regulation of their thoughts and feelings to achieve their goals. Therefore, social information processing and self-regulation continue as a cycle [60,61]. It is also emphasized in studies that children with advanced social information processing skills are more successful in self-regulation skills [62,63], as well as the effect of social context on self-regulation [59,64]. Focusing on the child’s emotions in the social information processing process, being aware of what the primary emotion is, and making predictions about behaviors and reactions depending on the situation may positively affect self-regulation. This is because self-regulation involves regulating one’s emotions and actions to achieve the goal [65], controlling impulses [66], and regulating emotions and actions according to situational needs and social norms.

To support the development of self-regulation skills, studies may need to be carried out to support social information processing in general and to reduce anger attribution in particular. In particular, ensuring impulse control has positive reflections on social interaction and relationships and can also enable the individual to look at the individual in a multidimensional way. The thoughts of a child with impulse control about which emotion they primarily feel and what they should do as a result may be related to more socially competent behaviors. In this respect, the child’s ability to control emotions and decide on reactions during the social information processing process may contribute to the development of self-regulation skills in the next stage. Considering that the processes of social information processing and self-regulation progress cyclically, each result is the beginning of the next step. For this reason, supporting the social information processing process will enable the individual to be more successful in self-regulation skills.

**H3.**  *Social information processing significantly affects the theory of mind through metacognition and self-regulation.*

The results show that social information processing (except STEP-P.P.B, one of the sub-dimensions of the STEP-P scale) has a significant effect on theory of mind through metacognition and self-regulation. In the study, it was found that social information processing has an effect on the theory of mind. This effect is through Metacognition and Self-Regulation. In the path analysis, the STEP-P test (except STEP-P.P.B), affected the theory of mind (TOMTB). This hypothesis is also consistent with previous research. The stage of the social information processing model that is most relevant to ToM is the representation stage, that is, the interpretation of cues. Attributing intentions and understanding others’ thoughts are representational skills, and it is hypothesized that children who show hostile intentions in ambiguous situations have difficulty understanding other people’s mental and emotional states [67]. In a longitudinal study examining emotional understanding, ToM, and pro-social orientation, it was found that emotional understanding, sympathy, ToM, and pro-social orientation were positively related [68]. Again, in a study examining ToM and peer relations in preschool children, it was found that aggression towards peers, exclusion by peers, and hyperactivity behaviors decreased as the ToM scale achievement increased [69]. In another study, it is stated that preschool children who exhibit aggressive peer interactions also have lower levels of self-regulation and ToM [70].

With the social information processing process, the individual first focuses on social clues then becomes aware of themself, turns towards both themself and their environment, and reaches a theory of mind. Theory of mind is related to the individual’s awareness of both their own and other individual’s thoughts and behaviors. As the actions and behaviors of others are made sense of, mental states begin to be better understood. In addition, interactions in social life also play a decisive role in the development of theory of mind. When evaluated in this context, the effect of social information processing on ToM may emerge. Also, in the related literature [71,72,73], it is seen that theory of mind is also related to interpreting actions and making sense of the cause–effect relationship, making moral judgements and making sense of emotional reactions. Therefore, social information processing is thought to have an indirect effect on the theory of mind.

Theory of mind is an umbrella term used to investigate the ability to attribute mental states to others. Therefore, metacognition, which is generally defined as ‘knowing what one knows’, is a part of the theory of mind [74] (p. 9). Metacognitive knowledge is a part of the theory of mind [75] (p. 167). It is also included in the literature that metacognition has a positive effect on ToM [76], metacognition and ToM are interrelated [29,77], and metacognition is both a concept related to ToM and a skill in ToM [74,75,78,79]. While metacognition is the realization of one’s thinking, the theory of mind is a process related to the understanding that one’s thinking is different from the thinking of others. Therefore, metacognition is thought to be an important factor in the development of an individual’s theory of mind. The development of the theory of mind is also an important concept for the comprehensibility of actions in daily life. Because metaphorical discourses, jokes, and insinuations can be understood through the theory of mind, these can be important in ensuring the continuity of social interaction and communication and for the individual to feel belonging to the environment in which they are in. The fact that metacognition and theory of mind focus on both other individuals and on oneself also enables these concepts to be interpreted about social and cognitive development. For this reason, the necessity of a holistic perspective in supporting the theory of mind is also proven by studies.

Social information processing (except STEP-P.P.B, one of the sub-dimensions of the STEP-P scale) has a significant effect on ToM through self-regulation. Self-regulation and theory of mind are interrelated concepts. Preschool years are an important developmental period in self-regulation skills. As preschool children mature, they become increasingly adept at softening or delaying their responses according to the demands of the situation, suppressing their responses completely when necessary, and shifting their attention away from salient distractors in contexts that require selective attention. There were also notable changes in the theory of mind during this period. The effects of self-regulation are seen not only in the emergence of theory of mind but also in the expression of theory of mind, and self-regulation is recognized as a very important facilitating factor for the development of theory of mind rather than just a “performance” factor. It is also emphasized that self-regulation is necessary but by no means sufficient for children to progress in ToM [80]. Studies [28,30,81] also emphasize that there is a relationship between self-regulation and ToM. Accordingly, it is seen that self-regulation plays a decisive role in ToM. Self-regulation skills, which are associated with the regulation of behavior, emotion, and thought, are thought to be an important factor in the development of the theory of mind, as they enable the individual to turn towards themself and become aware of themself. Self-regulation is included in the development of the theory of mind in terms of enabling the individual to become aware of and regulate themself and to focus on the behaviors of other individuals. This framework may also provide a perspective on the necessity of self-regulation in the development of the theory of mind.

## 5. Conclusions and Recommendations

According to the results obtained in the study, the ToM skills of 3–5-year-old children are affected by social information processing, metacognition, and self-regulation. For social information processing, the child’s use of cooperative and aggressive behaviors, attitudes, and strategies to achieve their goal, their interpretations of the reasons for the behaviors, and the emotions that the behavior will make the person feel affect metacognition. At the same time, in the process of social information processing, determining the reactions of children regarding the reactions of anger towards the events and the expected behaviors and reactions depending on the events is effective on self-regulation. Self-regulation, which enables individuals to control their behaviors, thoughts, and emotions, is also effective in the development process of the theory of mind. Metacognition, which is related to the individual’s self-awareness and their thoughts about what, why, and how they do what they do, also affects the theory of mind and supports its development. In this context, the development of the theory of mind includes concepts that are interconnected or have the power to influence each other.

Based on these results, social information processing should be supported to support the development of ToM. For this, children’s reactions should be well observed in social environments and children should be able to assume the positive or negative consequences of their behaviors. For the development of self-regulation and metacognition, which have a direct impact on the development of the theory of mind, children should be able to control their behaviors, thoughts, and emotions, and focus on what they are doing, why they are doing it, the facilitating or complicating factors, etc., rather than automatization or rote behavior. A suitable supportive educational environment can be created for the child to gain this process of awareness. Because the interactive environment, positive communication, and language-rich use of language in the home and school environment play an important role in the child’s ToM development. For this reason, it is recommended that there should be qualified sharing and information transfer both in the school and home environment. Further research can be planned with skills such as language, executive functions, empathy, and moral judgments, which are thought to be effective on ToM.

This study differs from other studies in that it is the first study to test the effect of social information processing, metacognition, and self-regulation on the development of ToM on the designed model, and it also provides practical and theoretical implications.

This study offers an alternative path for educators working in the field of special education. The development of theory of mind in individuals with special needs is more difficult. Educators working in the field of special education will see the necessity of working with social information processing, metacognition, and self-regulation variables to support ToM in individuals with special needs, and they will be able to support the development of ToM in children with special needs more comfortably and systematically. It is thought that educators working with individuals with autism spectrum disorder can also use this model. It is thought that sequential activities that can be performed on the way from social information processing to theory of mind will make the process more concrete, so that theory of mind can be supported more easily.

With this study, educators will understand how to organize a quality classroom environment and which goals should be set to support ToM (such as supporting social information processing first). At the same time, educators will be clearer in organizing the educational environment and plan.

With this study, parents will be able to be more selective in the language they use in daily life while supporting their children, use communication methods that will reveal how children perceive emotions, behaviors, and actions, and create environments that will enable their children to notice their own and other people’s emotions, behaviors, and actions.

## 6. Limitations

This study, which is a first in its field, differs from other studies by examining social information processing, metacognition, and self-regulation, which have an impact on the development of theory of mind, with path analysis, one of the types of structural equation modeling. However, this study is not without limitations. These limitations will be enlightening for future research. First of all, this study was conducted with cross-sectional data. Therefore, future research can plan a study with a longitudinal or experimental model.

The second limitation of this study is that the sample was conducted with 3–5-year-old children attending kindergartens and preschools affiliated with the Ministry of National Education in Konya city center in Turkey. Future research could try to extend, complement, or create an alternative model by analyzing the model used in this study with 3–5-year-old children in different cultures.

The third limitation of this study is that only social information processing, metacognition, and self-regulation, which are considered to be effective on ToM, were included in the model. Future research may include skills such as empathy, language, and executive functions that are effective on ToM or test a new model. In this way, a more comprehensive and extended model can be created.

The fourth limitation of this study is that there are limited studies in the literature on social information processing, metacognition, self-regulation, and ToM. Future research can test the developmental stage from social information processing skills to ToM in different age groups (e.g., adolescence, middle childhood, adulthood) using the current model. This may provide additional information about the reliability of the model.

## Figures and Tables

**Table 1:** Descriptive statistics on the personal characteristics of the participants.

	Statistics
**Age**	
Mean ± SD	55.95 ± 8.28
M (min–max)	58 (37–70)
**Age category**	
30–39 years	7 (2.3%)
40–49 years	68 (21.9%)
50 years and older	235 (75.8%)
**Gender**	
Female	151 (48.7%)
Male	159 (51.3%)
**Birth order**	
First child	160 (51.6%)
Middle child or one of the middle children	38 (12.3%)
Last Child	112 (36.1%)
**Number of children**	
1 child	72 (23.2%)
2 children	164 (52.9%)
3 children	56 (18.1%)
4 children and more	18 (5.8%)
**Duration of attendance**	
0–6 months	161 (51.9%)
7–12 months	47 (15.2%)
13–18 months	35 (11.3%)
19–24 months	36 (11.6%)
More than two years	31 (10%)
**Economic situation**	
Low	8 (2.6%)
Medium	272 (87.7%)
High	30 (9.7%)

Summary statistics are given as mean ± standard and Median (minimum. maximum) for numerical data and number (percentage) for categorical data.

**Table 2:** Descriptive statistics of the characteristics of the participants’ parents.

	Statistics
**Mother’s age**	
29 years and below	70 (22.6%)
30–39 years	198 (63.9%)
40–49 years	42 (13.5%)
**Father’s age**	
29 years and below	30 (9.7%)
30–39 years	191 (61.6%)
40–49 years	81 (26.1%)
50 years and older	8 (2.6%)
**Mother’s Education**	
Primary and secondary school	36 (11.6%)
High School	53 (17.1%)
University	196 (63.2%)
Postgraduate	25 (8.1%)
**Father’s Education**	
Primary and secondary school	21 (6.8%)
High School	50 (16.1%)
University	189 (61%)
Postgraduate	50 (16.1%)
**Mother’s employment status**	
Working	162 (52.3%)
Not working	148 (47.7%)
**Father’s employment status**	
Working	304 (98.1%)
Not working	6 (1.9%)
**Mother’s occupation**	
Housewife	132 (42.6%)
Officer	79 (25.5%)
Worker	6 (1.9%)
Self-employed	5 (1.6%)
Other	88 (28.4%)
**Father’s occupation**	
Officer	86 (27.7%)
Worker	22 (7.1%)
Self-employed	70 (22.6%)
Other	132 (42.6%)

Summary statistics are given as number (percentage) values.

**Table 3:** The mediating effect of MCKI and OZ scores on the effect of STEP-P scores on TOMTB.

Model	Dependent	Independent	*β* (LLCI; ULCI)	*R^2^*	*F*
Model 1	TOMTB	STEP-P.D1	**0.888 (0.518; 1.257)**	0.07	**22.356**
	MCKI	STEP-P.D1	**0.262 (0.087; 0.437)**	0.03	**8.630**
	OZ	STEP-P.D1	**1.162 (0.886; 1.438)**	0.18	**68.508**
	TOMTB	STEP-P.D1	**0.480 (0.103; 0.857)**	0.21	**27.233**
		MCKI	**0.686 (0.460; 0.913)**		
		OZ	**0.196 (0.052; 0.340)**		
Model 2	TOMTB	STEP-P.D2	**−1.084 (−1.485; −0.684)**	0.08	**28.420**
	MCKI	STEP-P.D2	**−0.325 (−0.516; −0.134)**	0.04	**11.237**
	OZ	STEP-P.D2	**−1.586 (−1.869; −1.303)**	0.28	**121.890**
	TOMTB	STEP-P.D2	**−0.601 (−1.041; −0.161)**	0.21	**27.631**
		MCKI	**0.689 (0.463; 0.915)**		
		OZ	**0.163 (0.011; 0.316)**		
Model 3	TOMTB	STEP-P.H1	**1.840 (1.389; 2.290)**	0.17	**64.584**
	MCKI	STEP-P.H1	**0.817 (0.605; 1.028)**	0.16	**57.828**
	OZ	STEP-P.H1	**0.847 (0.463; 1.231)**	0.06	**18.841**
	TOMTB	STEP-P.H1	**1.260 (0.791; 1.730)**	0.26	**36.180**
		MCKI	**0.478 (0.244; 0.711)**		
		OZ	**0.223 (0.095; 0.352)**		
Model 4	TOMTB	STEP-P.H2	**−0.632 (−1.189; −0.074)**	0.02	**4.971**
	MCKI	STEP-P.H2	**−0.374 (−0.632; −0.116)**	0.03	**8.143**
	OZ	STEP-P.H2	−0.038 (−0.487; 0.411)	0.00	0.028
	TOMTB	STEP-P.H2	−0.371 (−0.883; 0.141)	0.20	**25.480**
		MCKI	**0.670 (0.439; 0.900)**		
		OZ	**0.273 (0.141; 0.406)**		
Model 5	TOMTB	STEP-P.H3	**−1.486 (−2.281; −0.691)**	0.04	**13.526**
	MCKI	STEP-P.H3	**−1.055 (−1.413; −0.696)**	0.10	**33.496**
	OZ	STEP-P.H3	**−1.004 (−1.643; −0.366)**	0.03	**9.579**
	TOMTB	STEP-P.H3	−0.536 (−1.307; 0.234)	0.20	**25.413**
		MCKI	**0.652 (0.415; 0.889)**		
		OZ	**0.261 (0.128; 0.395)**		
Model 6	TOMTB	STEP-P.P.A	**1.552 (1.083; 2.020)**	0.12	**42.483**
	MCKI	STEP-P.P.A	**0.691 (0.472; 0.910)**	0.11	**38.600**
	OZ	STEP-P.P.A	**1.078 (0.698; 1.458)**	0.09	**31.118**
	TOMTB	STEP-P.P.A	**0.923 (0.444; 1.401)**	0.23	**30.598**
		MCKI	**0.579 (0.348; 0.811)**		
		OZ	**0.212 (0.079; 0.345)**		
Model 7	TOMTB	STEP-P.P.B	**−0.555 (−0.906; −0.205)**	0.03	**9.717**
	MCKI	STEP-P.P. B	−0.120 (−0.285; 0.045)	0.01	2.056
	OZ	STEP-P.P. B	−0.053 (−0.337; 0.231)	0.00	0.135
	TOMTB	STEP-P.P. B	**−0.460 (−0.778; −0.143)**	0.22	**28.007**
		MCKI	**0.671 (0.445; 0.898)**		
		OZ	**0.270 (0.139; 0.401)**		
Model 8	TOMTB	STEP-P.P.C	**1.040 (0.644; 1.436)**	0.08	**26.716**
	MCKI	STEP-P.P.C	**0.580 (0.400; 0.761)**	0.11	**39.952**
	OZ	STEP-P.P.C	**0.574 (0.251; 0.898)**	0.04	**12.221**
	TOMTB	STEP-P.P.C	**0.549 (0.157; 0.942)**	0.21	**27.779**
		MCKI	**0.599 (0.362; 0.835)**		
		OZ	**0.251 (0.119; 0.383)**		
Model 9	TOMTB	STEP-P.P.D	**1.963 (1.507; 2.419)**	0.19	**71.765**
	MCKI	STEP-P.P.D	**0.531 (0.304; 0.759)**	0.06	**21.079**
	OZ	STEP-P.P.D	**0.945 (0.554; 1.335)**	0.07	**22.695**
	TOMTB	STEP-P.P.D	**1.489 (1.038; 1.940)**	0.29	**42.126**
		MCKI	**0.563 (0.344; 0.781)**		
		OZ	**0.185 (0.058; 0.313)**		

*β*: regression coefficient, *R*^2^: coefficient of determination; bolded sections are statistically significant (*p* < 0.05).

**Table 4:** Effects between TOMTB, STEP-P, MCKI, and OZ model.

Dependent	Total Effect	Direct Effect	Indirect Effect		
			Total	MCKI	OZ
	*β* (LLCI; ULCI)	*β* (LLCI; ULCI)	*β* (LLCI; ULCI)	*β* (LLCI; ULCI)	*β* (LLCI; ULCI)
STEP-P.D1	**0.888** **(0.518; 1.257)**	**0.480** **(0.103; 0.857)**	**0.408** **(0.197; 0.647)**	**0.180** **(0.067; 0.316)**	**0.228** **(0.036; 0.447)**
STEP-P.D2	**−1.084** **(−1.485; −0.684)**	**−0.601** **(−1.041; −0.161)**	**−0.483** **(−0.772; −0.193)**	**−0.224** **(−0.361; −0.102)**	−0.259 (−0.551; 0.023)
STEP-P.H1	**1.840** **(1.389; 2.290)**	**1.260** **(0.791; 1.730)**	**0.579** **(0.352; 0.834)**	**0.390** **(0.180; 0.610)**	**0.189** **(0.056; 0.364)**
STEP-P.H2	**−0.632** **(−1.189; −0.074)**	−0.371 (−0.883; 0.141)	**−0.261** **(−0.515; −0.014)**	**−0.250** **(−0.461; −0.070)**	−0.011 (−0.137; 0.114)
STEP-P.H3	**−1.486** **(−2.281; −0.691)**	−0.536 (−1.307; 0.234)	**−0.950** **(−1.335; −0.577)**	**−0.687** **(−1.013; −0.388)**	**−0.263** **(−0.545; −0.064)**
STEP-P.P.A	**1.552** **(1.083; 2.020)**	**0.923** **(0.444; 1.401)**	**0.629** **(0.397; 0.893)**	**0.400** **(0.219; 0.611)**	**0.229** **(0.064; 0.436)**
STEP-P.P.B	**−0.555** **(−0.906; −0.205)**	**−0.460** **(−0.778; −0.143)**	−0.095 (−0.232; 0.039)	−0.081 (−0.188; 0.019)	−0.014 (−0.09; 0.056)
STEP-P.P.C	**1.040** **(0.644; 1.436)**	**0.549** **(0.157; 0.942)**	**0.491** **(0.304; 0.711)**	**0.347** **(0.189; 0.527)**	**0.144** **(0.035; 0.306)**
STEP-P.P.D	**1.963 ** **(1.507; 2.419)**	**1.489** **(1.038; 1.940)**	**0.474** **(0.277; 0.705)**	**0.299** **(0.156; 0.474)**	**0.175** **(0.033; 0.355)**

*β*: regression coefficient, bolded sections are statistically significant (*p* < 0.05).

## Data Availability

The raw data supporting the conclusions of this article will be made available by the authors on request.

## References

[1] Slaughter V., Repacholi B., Repacholi B., Slaughter V. (2003). Introduction: Individual differences in theory of mind what are we investigating?. Individual Differences in Theory of Mind: Implications for Typical and Atypical Development.

[2] Bjorklund D.F., Cormier C.A., Rosenberg J.S., Schneider W., Schumann-Hengsteler R., Sodian B. (2005). The evolution of theory of mind: Big brains, social complexity, and inhibition. Young Children’s Cognitive Development Interrelationships among Executive Functioning, Working Memory, Verbal Ability and Theory of Mind.

[3] Astington J.W., Dack L.A., Haith M.M., Benson J.B. (2008). Theory of mind. Encyclopedia Od Infant and Early Childhood Development Volume 1.

[4] Keçeli Kaysılı B. (2013). Theory of mind: A Comparison of children with autism spectrum disorders and typically developing children. Ank. Univ. Fac. Educ. Sci. Spec. Educ. J..

[5] Pelphrey K.A., Shultz S., Hudac C.M., Vander Wyk B.C. (2011). Research review: Constraining heterogeneity: The social brain and its development in autism spectrum disorder. J. Child Psychol. Psychiatry.

[6] Sodian B., Schneider W., Schumann-Hengsteler R., Sodian B. (2005). Theory of mind—The case for conceptual development. Young Children’s Cognitive Development Interrelationships among Executive Functioning, Working Memory, Verbal Ability, and Theory of Mind.

[7] Martinez M.E. (2006). What is metacognition?. Phi Delta Kappan.

[8] Garner R., Alexander P.A. (1989). Metacognition: Answered and unanswered questions. Educ. Psychol..

[9] Zimmerman B.J., Boekaerts M., Pintrich P.R., Zeidner M. (2000). Attaining self-regulation: A social cognitive perspective. Handbook of Self-Regulation.

[10] Dodge K.A., Rabiner D.L. (2004). Returning to roots: On social information processing and moral development. Child Dev..

[11] Crick N.R., Dodge K.A. (1994). A review and reformulation of social information-processing mechanisms in children’s social adjustment. Psychol. Bull..

[12] Cooke T. (2017). Social information processing: A useful framework for educational psychology. Educ. Psychol. Res. Pract..

[13] Ziv Y. (2013). Social information processing patterns, social skills, and school readiness in preschool children. J. Exp. Child Psychol..

[14] Lockl K., Schneider W. (2007). Knowledge about the mind: Links between theory of mind and later metamemory. Child Dev..

[15] Hughes C., Jaffee S.R., Happé F., Taylor A., Caspi A., Moffitt T.E. (2005). Origins of ındividual differences in theory of mind: From nature to nurture?. Child Dev..

[16] Ebert S. (2020). Theory of mind, language, and reading: Developmental relations from early childhood to early adolescence. J. Exp. Child Psychol..

[17] Fujino H., Fukushima K., Fujiyoshi A. (2017). Theory of mind and language development in japanese children with hearing loss. Int. J. Pediatr. Otorhinolaryngol..

[18] McElwain N.L., Ravindran N., Emery H.T., Swartz R. (2019). Theory of mind as a mechanism linking mother–toddler relationship quality and child–friend interaction during the preschool years. Soc. Dev..

[19] Witt S., Weitkämper A., Neumann H., Lücke T., Zmyj N. (2018). Delayed theory of mind development in children born preterm: A longitudinal study. Early Hum. Dev..

[20] Goffin K.C., Kochanska G., Yoon J.E. (2020). Children’s theory of mind as a mechanism linking parents’ mind-mindedness in infancy with children’s conscience. J. Exp. Child Psychol..

[21] Capage L., Watson A.C. (2001). Individual differences in theory of mind, aggressive behavior, and social skills in young children. Early Educ. Dev..

[22] Carlson S.M., Moses L.J. (2001). Individual differences in inhibitory control and children’s theory of mind. Child Dev..

[23] Carlson S.M., Moses L.J., Hix H.R. (1998). The role of ınhibitory processes in young children’s difficulties with deception and false belief. Child Dev..

[24] Carlson S.M., Moses L.J., Breton C. (2002). How specific is the relation between executive function and theory of mind? Contributions of ınhibitory control and working memory. Infant Child Dev..

[25] Mcinnis M.A. (2014). The Relation between Theory of Mind and Empathy in Preschool: The Case of Fantasy Orientation. Ph.D. Thesis.

[26] Goldstein T.R., Winner E. (2012). Enhancing empathy and theory of mind. J. Cogn. Dev..

[27] Bensalah L., Caillies S., Anduze M. (2016). Links among cognitive empathy, theory of mind, and affective perspective taking by young children. J. Genet. Psychol..

[28] Jahromi L.B., Stifter C.A. (2008). Individual differences in preschoolers’ self-regulation and theory of mind. Merrill-Palmer Q..

[29] Mohamed A.H.H. (2012). The relationship between metacognition and self-regulation in young children. Procedia-Soc. Behav. Sci..

[30] Özbek E. (2021). The Relationship between Self Regulation and Theory of Mind Skills among 60–72 Months Aged Children. Master’s Thesis.

[31] Etel E. (2012). Social Competence, Theory of Mind, and Executive Function in Institution-Reared turkish Preschoolers. Master’s Thesis.

[32] Gürleyik S. (2018). Theory of Mind and Peer Relationships in Pre-School Children. Master’s Thesis.

[33] Robson S. (2006). Developing Thinking and Understanding in Young Children an Introduction for Students.

[34] Sönmez V., Alacapınar F.G. (2018). Scientific Research Methods with Examples, Extended.

[35] Çokluk Ö., Şekercioğlu G., Büyüköztürk Ş. (2018). Multivariate Statistics for Social Sciences: SPSS and Lisrel Applications.

[36] Fritz M.S., MacKinnon D.P. (2007). Required sample size to detect the mediated effect. Psychol. Sci..

[37] MacKinnon D.P., Cheong J., Pirlott A.G., Cooper H., Camic P.M., Long D.L., Panter A.T., Rindskopf D., Sher K.J. (2012). Statistical mediation analysis. APA Handbook of Research Methods in Psychology, Vol. 2. Research Designs: Quantitative, Qualitative, Neuropsychological, and Biological.

[38] Qin X. (2024). Sample size and power calculations for causal medication analysis: A tutorial and Shiny App. Behav. Res. Methods.

[39] Hutchins T.L., Prelock P. (2010). Technical manual for the Theory of Mind Task Battery. Unpublished Copyrighted Manuscript. https://www.theoryofmindinventory.com.

[40] Marulis L.M., Palincsar A.S., Berhenke A.L., Whitebread D. (2016). Assessing metacognitive knowledge in 3–5 year olds: The development of a metacognitive knowledge interview (McKI). Metacogn. Learn..

[41] Schultz D., Ambike A., Logie S.K., Bohner K., Stapleton L.M., Vanderwalde H., Min C.B., Betkowski J.A. (2010). The development and validation of a videobased assessment of young children’s social information processing. J. Abnorm. Child Psychol..

[42] Fındık Tanrıbuyurdu E. (2012). Validity and Realibility Study of Preschool Self-Regulation Assessment. Master’s Thesis.

[43] Ullman J.B., Tabachnick B.G., Fidell L.S. (2013). Structural equation modeling. Using Multivariate Statistics.

[44] Preacher K.J., Rucker D.D., Hayes A.F. (2007). Addressing moderated mediation hypotheses: Theory, methods, and prescriptions. Multivar. Behav. Res..

[45] Zhao X., Lynch J.G., Chen Q. (2010). Reconsidering Baron and Kenny: Myths and truths about mediation analysis. J. Consum. Res..

[46] Preacher K.J., Hayes A.F. (2008). Asymptotic and resampling strategies for assessing and comparing indirect effects in multiple mediator models. Behav. Res. Methods.

[47] Karagöz Y. (2019). SPSS-AMOS-META Applied Statistical Analysis.

[48] Downing K., Ho R., Shin K., Vrijmoed L., Wong E. (2007). Metacognitive development and moving away. Educ. Stud..

[49] Baker L., Reese H.W. (1994). Fostering metacognitive development. Advances in Child Development and Behavior Volume 25.

[50] Reeve R.A., Brown A.L. (1985). Metacognition reconsidered: Implications for ıntervention research. J. Abnorm. Child Psychol..

[51] Larkin S. (2009). Metacognition in Young Children.

[52] Whitebread D., Bingham S., Grau V., Pasternak D.P., Sangster C. (2007). Development of metacognition and self-regulated learning in young children: Role of collaborative and peer-assisted learning. J. Cogn. Educ. Psychol..

[53] Cassata A.E., French L. Using concept mapping to facilitate metacognitive control in preschool children. Proceedings of the Theory, Methodology, Technology Proceedings of the Second International Conference on Concept Mapping.

[54] Son L.K., Kornell N., Finn B., Cantlon J.F., Briñol P., Demarree K.G. (2012). Metacognition and the social animal. Social Metacognition.

[55] Salonen P., Vauras M., Efklides A. (2005). Social interaction-what can it tell us about metacognition and coregulation in learning?. Eur. Psychol..

[56] Papleontiou Louca E. (2003). The concept and instruction of metacognition. Teach. Dev..

[57] Chiu M.M., Kuo S.W., Larsen C.B. (2009). Social metacognition in groups: Benefits, difficulties, learning and teaching. Metacognition: New Research Developments.

[58] Petlichkoff L.M. Self-Regulation Skills for Children and Adolescents. In *Developmental Sport and Exercise Psychology: A Lifespan Perspective*; 2004. https://www.researchgate.net/publication/288941092_Self-regulation_skills_for_children_and_adolescents.

[59] Lochman J.E., Koops W., Brugman D., Ferguson T.J., Sanders A.F. (2010). Social cognition and selfregulation: Change in outcome expectations and aggressive behaviour over time. The Development and Structure of Conscience.

[60] Dodge K.A., Perlmutter M. (1986). A Social ınformation processing model of social competence in children. Cognitive Perspectives on Children’s Social and Behavioral Development: The Minnesota Symposia on Child Psychology Volume 18.

[61] Usher E.L., Schunk D.H., Schunk D.H., Greene J.A. (2018). Social cognitive theoretical perspective of self regulation. Handbook of Self-Regulation of Learning and Performance.

[62] Öner Ş., Özbey S. (2022). Investigation of the relationship between social information processing skills and psychological resilience levels of preschool children. Int. J. Soc. Humanit. Adm. Sci..

[63] Şenol F.B., Metin E. (2021). Social information processing in preschool children: Relations to social interaction. Particip. Educ. Res..

[64] Morf C.C., Horvath S., Hoyle R.H. (2010). Self-regulation processes and their signatures dynamics of the self-system. Handbook of Personality and Self-Regulation.

[65] Schunk D.H. (2012). Learning Theories an Educational Perspective.

[66] Bodrova E.E., Leong D.J. (2008). Developing self-regulation in kindergarten can we keep all the crickets in the basket?. Young Child..

[67] Yağmurlu B. (2014). Relations among sociocognitive abilities and prosocial behavior. J. Child Fam. Stud..

[68] Eggum N.D., Eisenberg N., Kao K., Spinrad T.L., Bolnick R., Hofer C., Kupfer A.S., Fabricius W.V. (2011). Emotion understanding, theory of mind, and prosocial orientation: Relations over time in early childhood. J. Posit. Psychol..

[69] Gürleyik S., Gözün Kahraman Ö. (2019). Investigation of the relationship between theory of mind and peer relations in preschool children. H. U. J. Educ..

[70] Olson S.L., Lopez-Duran N., Lunkenheimer E.S., Chang H., Sameroff A.J. (2011). Individual differences in the development of early peer aggression: Integrating contributions of self-regulation, theory of mind, and parenting. Dev. Psychopathol..

[71] Astington J.W., Repacholi B., Slaughter V. (2003). Sometimes necessary, never sufficient false-belief understanding and social competence. Individual Differences in Theory of Mind: Implications for Typical and Atypical Development.

[72] Buon M., Seara-Cardoso A., Viding E. (2016). Why (and how) should we study the ınterplay between emotional arousal, theory of mind, and ınhibitory control to understand moral cognition?. Psychon. Bull. Rev..

[73] Wellman H.M. (1990). The Child’s Theory of Mind.

[74] Beran M.J., Brandl J.L., Perner J., Proust J., Beran M.J., Brandl J.L., Perner J., Proust J. (2012). On the nature, evolution, development, and epistemology of metacognition: Introductory thoughts. Foundations of Metacognition.

[75] Kloo D., Rohwer M., Beran M.J., Brandl J.L., Perner J., Proust J. (2012). The development of earlier and later forms of metacognitive abilities: Reflections on agency and ignorance. Foundations of Metacognition.

[76] Aydın U., Özgeldi M. (2019). Unpacking the roles of metacognition and theory of mind in Turkish undergraduate students’ academic achievement: A test of two mediation models. Croat. J. Educ..

[77] Feurer E., Sassu R., Cimeli P., Roebers C. (2015). Development of meta-representations: Procedural metacognition and the relationship to theory of mind. J. Educ. Dev. Psychol..

[78] Schneider W., Lockl K., Fernandez O., Schneider W., Schumann-Hengsteler R., Sodian B. (2005). Interrelationships among theory of mind, executive control, language development, and working memory in young children: A Longitudinal analysis. Young Children’s Cognitive Development Interrelationships among Executive Functioning, Working Memory, Verbal Ability, and Theory of Mind.

[79] Efklides A., Misailidi P., Efklides A., Misailidi P. (2010). Introduction: The present and the future in metacognition. Trends and Prospects in Metacognition Research.

[80] Moses L.J., Carlson S.M., Lightfoot C., Lalonde C., Chandler M. (2004). Self-regulation and children’s theories of mind. Changing Conceptions of Psychological Life.

[81] Vithlani P.P. (2010). Emotion Regulation and Executive Functioning as Predictors of Theory of Mind Competence during Early Childhood. Master’s Thesis.

